# Hemotoxic effects of polyethylene microplastics on mice

**DOI:** 10.3389/fphys.2023.1072797

**Published:** 2023-03-08

**Authors:** Souzan Abdel-Zaher, Mahmoud S. Mohamed, Alaa El-Din H. Sayed

**Affiliations:** ^1^ Department of Molecular Biology, Molecular Biology Researches and Studies Institute, Assiut University, Assiut, Egypt; ^2^ Zoology Department, Faculty of Science, Assiut University, Assiut, Egypt

**Keywords:** mice, PE-MPs, erythrocytes, helmet cells, liver functions

## Abstract

Micro- or nanoplastics, which are fragmented or otherwise tiny plastic materials, have long been a source of environmental worry. Microplastics (MPs) have been well documented to alter the physiology and behavior of marine invertebrates. The effects of some of these factors are also seen in larger marine vertebrates, such as fish. More recently, mouse models have been used to investigate the potential impacts of micro- and nanoplastics on host cellular and metabolic damages as well as mammalian gut flora. The impact on erythrocytes, which carry oxygen to all cells, has not yet been determined. Therefore, the current study aims to ascertain the impact of exposure to various MP exposure levels on hematological alterations and biochemical indicators of liver and kidney functions. In this study, a C57BL/6 murine model was concentration-dependently exposed to microplastics (6, 60, and 600 μg/day) for 15 days, followed by 15 days of recovery. The results demonstrated that exposure to 600 μg/day of MPs considerably impacted RBCs’ typical structure, resulting in numerous aberrant shapes. Furthermore, concentration-dependent reductions in hematological markers were observed. Additional biochemical testing revealed that MP exposure impacted the liver and renal functioning. Taken together, the current study reveals the severe impacts of MPs on mouse blood parameters, erythrocyte deformation, and consequently, anemic patterns of the blood.

## 1 Introduction

Plastic particles with a diameter of less than 5 mm are now widely acknowledged as a threat to the environment and a health risk to human populations, ranging from oxidative stress to DNA damage (Rochman et al., 2013; Katsnelson 2015; Smith et al., 2018; Prata et al., 2020; Ibrahim et al., 2021; Blackburn and Green 2022). There are two major sources of microplastics (MPs):

1) cosmetics, detergents, sunscreens, and medicine delivery systems all containing plastic powders or particles (Galloway 2015) and
2) bigger plastic pieces breaking down in the environment due to UV radiation, mechanical abrasion, and biological deterioration (Andrady 2011).

MPs have been found in various settings and media, such as rivers, sewage, sediments, soil, and even table salt (Rillig 2012; Imhof et al., 2013; Jabeen et al., 2015; Lechner and Ramler 2015; Lusher et al., 2015).

MPs can reach human populations either directly through the environment or indirectly through food. According to numerous studies (Thompson et al., 2009; Cole et al., 2013) and food chains (Setälä et al., 2014), a variety of marine organisms (bivalves, fish, etc.) consume MPs. MPs are therefore anticipated to accumulate in the environment and increase the risk of exposure for wild creatures and human populations over time due to their extensive use and durability.

Studies demonstrating the potential health risk and tissue accumulation of MPs in mammals are scarce, although most research on the toxic effects of MPs has been on aquatic creatures. Accumulation of MPs in tissues can have various negative effects, which include physical harm. This is because the majority of these plastics have been detected in the marine animals examined, and it has been hypothesized that malnutrition contributes to their death, which is the rupture of the stomach from being trapped by debris (De Stephanis et al., 2013). Many species of birds, reptiles, and fish were found to directly ingest plastic, which may block their stomach and intestines (Hämer et al., 2014). This may cause inhibition of growth and development (Koelmans et al., 2014) and energy deficiency (Galloway 2015). Among the cellular impacts were modifications to immunological responses, the lysosomal compartment, peroxisome proliferation, antioxidant system, neurotoxic effects, and the start of genotoxicity (Avio et al., 2015; Hamed et al., 2019; Hamed et al. 2020; Hamed et al. 2021; Sayed et al., 2021; Ammar et al., 2022; Sayed et al., 2022; Sayed et al., 2022). Microplastic-induced reactive oxygen species (ROS) has been shown to be an inducer of oxidative stress in some marine organisms (Wright et al., 2013). It has been found to have severe effects on the feeding and water behavior as well as the metabolism of fish. Hence, it has been concluded that polystyrene nanoparticles have severe effects on both behavior and metabolism (Mattsson et al., 2015). Therefore, information on MP tissue accumulation in mammalian models would be crucial for determining the risk of MPs to human health (Prata et al., 2020; Ibrahim et al., 2021; Blackburn and Green 2022).

The toxic effects of MPs on erythrocytes (RBCs) are yet to be verified. Hence, the current study determined how exposure to different MP concentrations influenced haematological changes and biochemical indicators of liver and kidney function as a proxy for the effects that microplastics on human health.

## 2 Materials and methods

### 2.1 Animals

This study used 60 C57BL/6 male mice purchased from the Tudor Institute in Cairo. They ranged in age from 2 months (Li et al., 2020) and were 20 g in weight. We divided the animals into four groups (15 mice in each group). At the Molecular Biology Research and Studies Institute, Assiut University, the practice was carried out in accordance with the established ethics regulations in the care and treatment of animals. The animals were housed at a temperature of 23 ± 2°C with a 12:12 light:dark cycle. Feed and drinking water were freely available to all animals.

### 2.2 Chemicals

Microplastics (MPs) were purchased from Toxemerge Pty Ltd. in Australia as powders with asymmetrical particles (>90% of the microplastics were larger than 100 nm in size). A methodology for characterizing the microplastics was performed using light and transmission electron microscopy at TEMU, Assiut University (Hamed et al., 2019).

### 2.3 Stock preparation and characterization for microplastics

A stock solution was prepared after the manufacturing procedure and kept at room temperature. Before each use, the stock solution (0.1 g MP/500 ml D.W.) was prepared by using a magnetic stirrer. From this stock, 30 μl representing a concentration of 6 μg, 300 µl representing 60 μg, and 3 ml representing 600 μg were taken just before the start of each experiment. MP particles were characterized using a light microscope.

### 2.4 Experimental approach

Fifteen mice each were assigned to one of four groups: group 1, which is considered the control group; group 2, which got 6 μg/ml of the MPs extract each day orally; group 3, which got 60 μg/ml of MPs; and group 4, which got 600 μg/ml of MPs, for 15 days (Li et al., 2020; Ragusa et al., 2021; Huang et al., 2022; Pironti et al., 2023).

### 2.5 RBC’s alterations

The blood was drawn, and smears were dried, fixed in 100% methanol for 10 min, and then stained with hematoxylin and eosin. Slides were chosen based on their staining quality and randomly graded to maintain anonymity. According to Al-Sabti and Metcalfe (1995), 3,000 cells (minimum of 100 cells per slide) were analyzed in each group under a ×40 objective to identify morphologically changed red blood corpuscles. The morphological changes of erythrocytes, which included acanthocytes, sickle-shaped cells, crenated cells, enlarged cells, and changes in nuclear morphology, were noted by using a VE-T2 microscope and were photographed using a 14 MP OMAX Camera (MN: A35140U3, China).

### 2.6 Hematological and biochemical parameters

Using an automatic hematology analyzer Mindary B. 2000, hematological and biochemical parameters such as RBCs, WBCs, differential WBCs, blood platelets, hematocrit (HCT), hemoglobin (Hb), mean corpuscular volume (MCV), mean corpuscular hemoglobin (MCH), and mean corpuscular hemoglobin concentration (MCHC), as well as glucose, total protein, and lymphocyte/neutrophil rates were analyzed.

### 2.7 Qualitative examination of microplastics

To identify MPs qualitatively and ascertain their presence in the digestive system, small, medium, and large intestinal fragments were cut and placed in a hydrogen peroxide solution (30%) before placing them in a hot water bath set to 70^°^C degrees for 2 hours.

The fourth group was observed to have more MPs than the second and third groups, which were also detected in these groups but in reduced amounts.

### 2.8 Statistic evaluation

The minimum, maximum, averages, standard errors, and measured parameter ranges are considered to constitute fundamental statistics. For the raw data, the homogeneity of variance was assumed. Additionally, one-way ANOVA was used to record the pattern of differences in all treatments and the control group in the absence of interactions. The tests of Tukey and Dunnett were considered for multiple comparisons. At a significance level <0.05, the IBM-SPSS software version 21 (IBM-SPSS 2012.) and Xls sheets were considered.

## 3 Results

### 3.1 Quantification and characterization of microplastics

**FIGURE 1:**
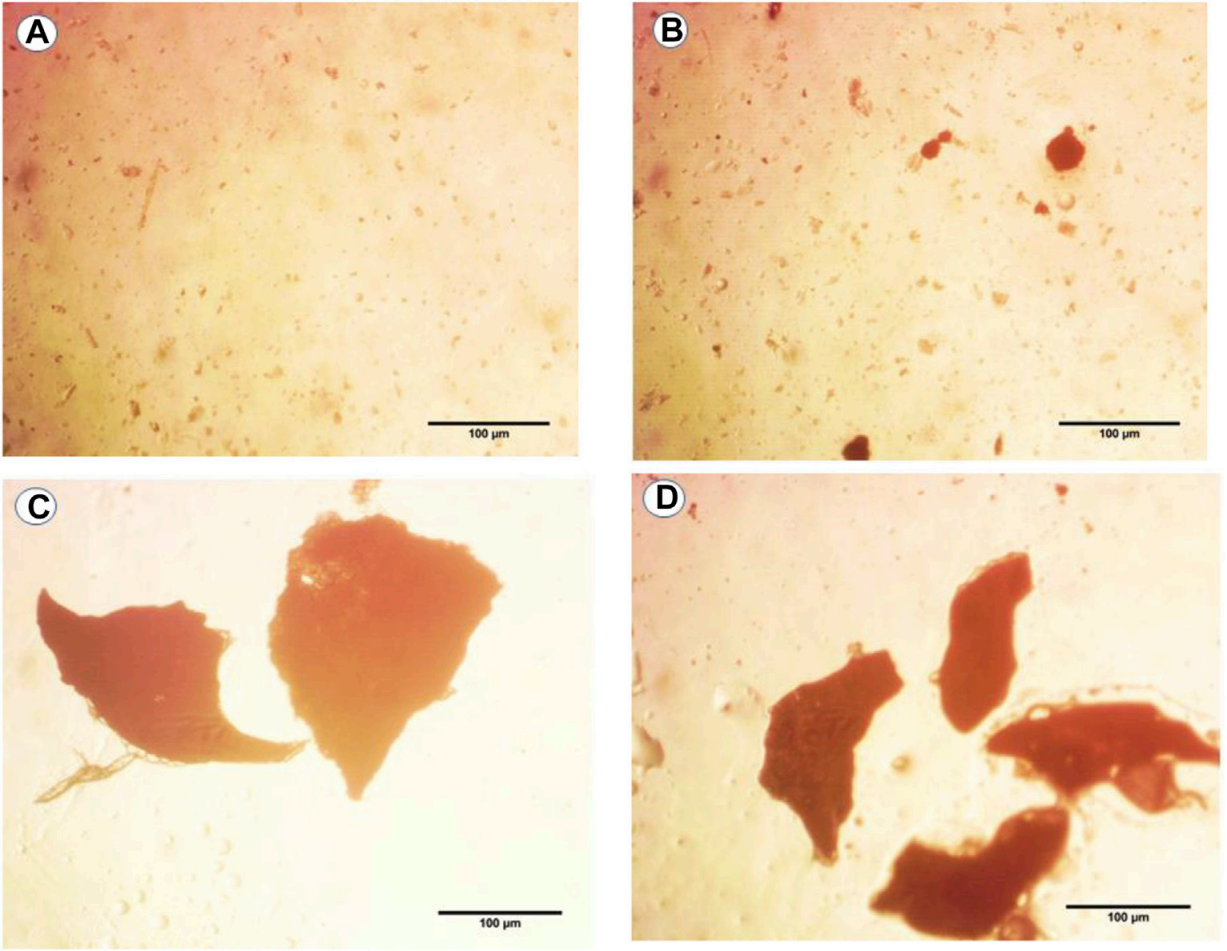
Photographs of light images of microplastics (MPs) found in dissolved mouse tissues in hydrogen peroxide solution after exposure to **(A)** control, **(B)** 6 μg/ml MPs, **(C)** 60 μg/ml MPs, and **(D)** 600 μg/ml MPs.

The pictures taken using the light microscope at a magnification of ×40 revealed that the microplastic particles were of irregular shapes (Figure 1). When compared to the control, the treated groups had more MPs in their guts. In contrast to the exposure groups, MPs were not found following the recovery period.

### 3.2 Erythrocytes alterations

**FIGURE 2:**
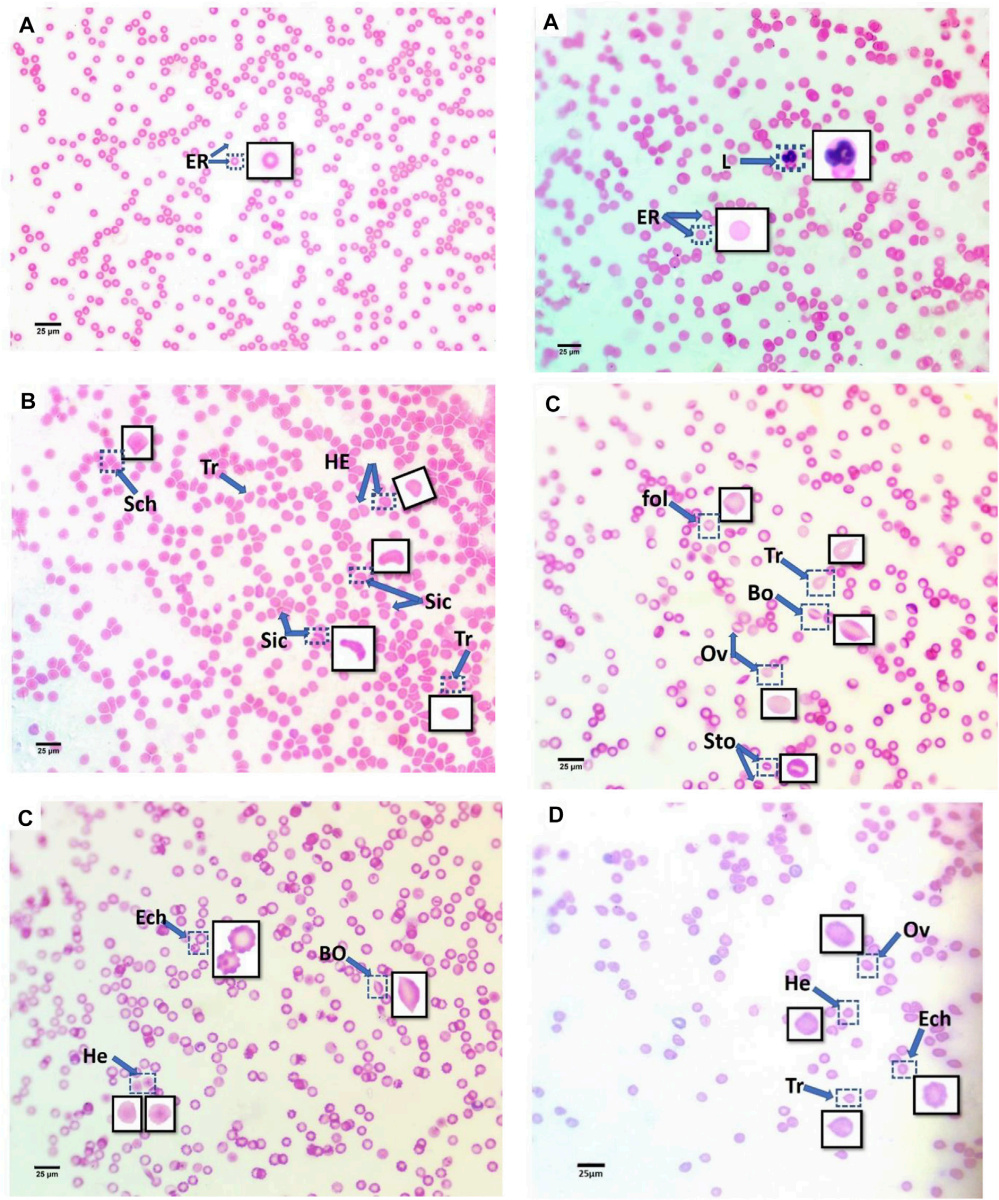
Blood smears of treatment mice groups with polyethylene microplastics: **(A)** control, **(B)** 6 μg/ml MPs, **(C)** 60 μg/ml MPs, and **(D)** 600 μg/ml MPs. Er: erythrocytes; L: leucocytes; Tr: teardrop-like cells; HE: helmet cell; Sto: stomatocytes; Sic: sickle cells; Sch: schistocytes; fol: folded cells; Bo: boat-shaped cell; Ov: ovalocytes; and Ech: echinocytes (H&E stained).

The blood smears of mice from all groups, stained with hematoxylin and eosin, are displayed in Figure 2. The blood smear in the control group represents the erythrocytes’ typical structure. According to Figure 2A, the blood comprises rounded, biconcave, non-nucleated erythrocytes (Er) and various leucocyte types (L).

In this study, the erythrocytes from the animals treated with MPs displayed various morphological patterns of malformed cells. As shown in Figure 2, these patterns include the teardrop-like cells (Pollastro and Pillmore, 1987), helmet cells (HE), sickle cells (Sic), schistocytes (Sch), folded cells (Gregorio et al., 2009), boat-shaped cells (Bo), ovarian cells (Ov), and echinocytes (Lechner and Ramler).

**FIGURE 3:**
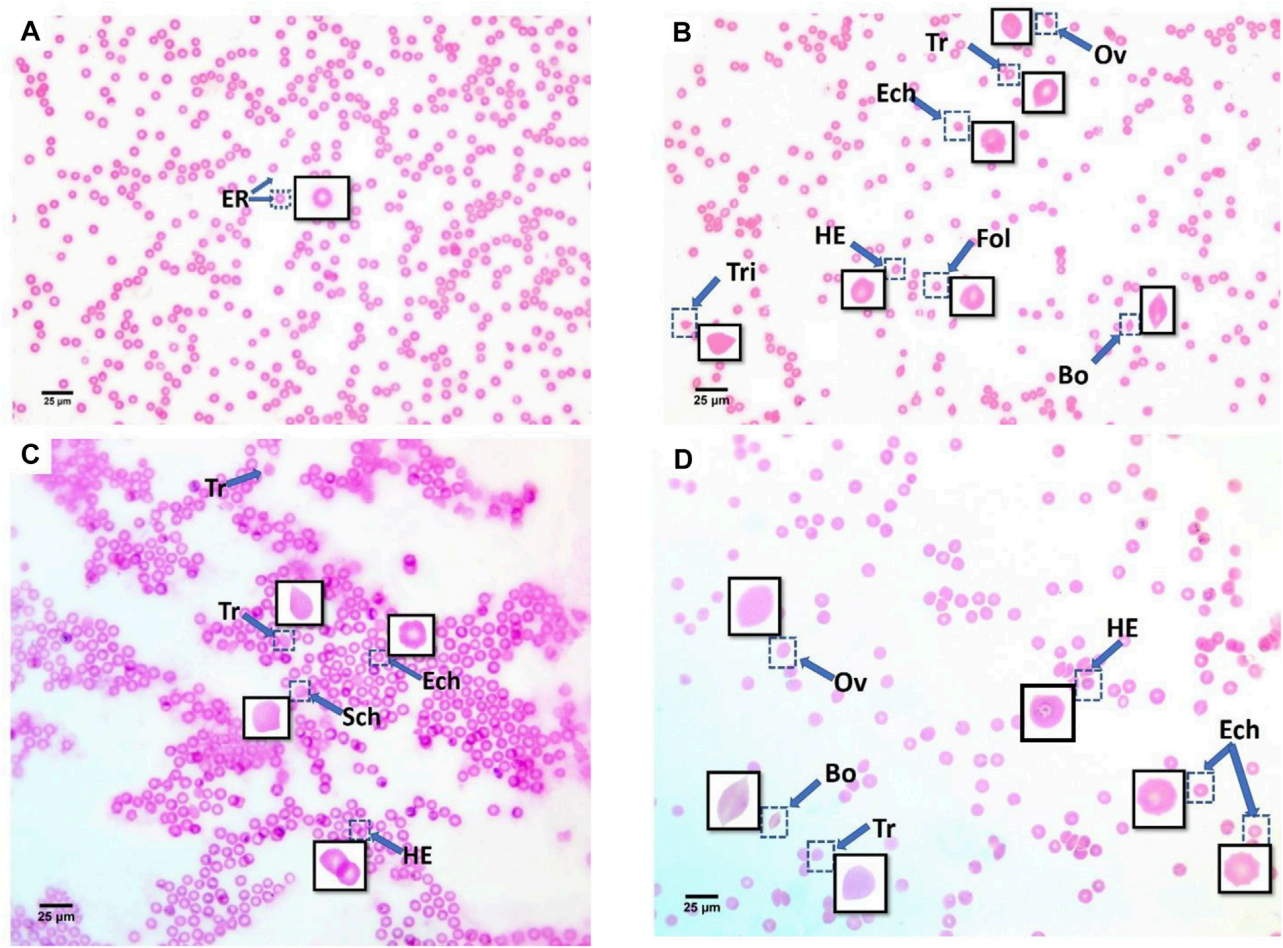
Blood smears of recovery mice groups with polyethylene microplastics: **(A)** control, **(B)** 6 μg/ml MPs, **(C)** 60 μg/ml MPs, and **(D)** 600 μg/ml MPs. ER: erythrocytes; Tr: teardrop-like cells; HE: helmet cell; Sic: sickle cells; Sch: schistocytes; fol: folded cells; Bo: boat-shaped cell; Ov: ovalocytes; Ech: echinocytes; and Tri: triangular cells (H&E stained).

Additionally, echinocytes exhibited the most specific change in red blood cell morphology that was noticed (Lechner and Ramler)—an indication of uremia—showing a significant increase to 13 in the 60 μg/ml MPs group, which spontaneously recovered after 15 days to 1.6. helmet cells (HE) that significantly increased to 7.9 in the 6 μg/ml MPs group, which also spontaneously recovered after 15 days to 0.033. Teardrop-like cells (Pollastro and Pillmore, 1987), which are considered indicators of myelofibrosis, showed a significant increase to 4.9 in the 6 μg/ml MPs group, which spontaneously recovered after 15 days to 1.5. It also showed a significant increase to 1.86 in the 60 μg/ml MPs group, which spontaneously recovered after 15 days to 0.6 (Figure 3; Table 1).

**TABLE 1:** Effect of 15 days of exposure to microplastics (MPs) and recovery on the blood alterations of the C57BL/6 mouse model. Data are represented as means ± SE. Values with different superscript letters in the same row for each parameter are significantly different (*p* < 0.05). Values of recovery period with * are significantly different from the exposure period.

Treatment	Boat-shaped cells	Teardrop-shaped cells	Schistocytes	Helmet cells	Ovalocytes	Triangular cells	Folded cells	Sickle cells	Echinocytes	Stomatocytes	Keratocytes	SC poikilocytes
Control	0.2 ± 0.1114^a^	1.4 ± 0.30924^a^	0^a**^	0^a**^	0.0667 ± 0.0667^a^	0^a**^	0^a**^	0.0667 ± 0.04632^a^	5.7 ±	0^a**^	0^a**^	0^a**^
									1.75129^a^			
Control R	0.3667± 0.1761^a^	2.0667 ± 0.2874^a^	0.0333 ± 0.0333^a*^	0.6 ± 0.20115^a^	0.4 ± 0.1768^a^	0^a**^	0.0333 ± 0.03333^a*^	0^a**^	0.1333± 0.09264^a^	0^a**^	0^a**^	0^a**^
6 µg/ml MP	0.6± 0.2738^a^	4.9333 ± 0.8427^b^	8.2 ± 1.2725^b^	7.9 ± 1.37661^b^	3.6333 ±1.0399^b^	0^a**^	0^a**^	1.2333 ± 0.35778^b^	0.2333 ± 0.1143a	0^a**^	1.8333 ± 0.6452^b^	0.2333± 0.1492^b^
6 µg/ml MPR	0.4 ± 0.1768^a^	1.5333 ± 0.2235^a^	0^a**^	0.0333 ± 0.0333^a*^	0.0667 ± 0.0463^a^	0.0333 ± 0.0333^a*^	0.0667± 0.04632^a^	0^a**^	1 ± 0.51862^a^	0^a**^	0^a**^	0^a**^
60 µg/ml MP	1.8333 ± 0.5298^b**^	1.8667 ± 0.5158^a^	0.0333 ± 0.0333^a*^	0.4333 ± 0.2864^a^	0.3667 ± 0.1825^a^	0.5± 0.2287^b^	0.6± 0.27376^b^	0.0333 ± 0.03333^a*^	13 ±	0.2 ± 0.1213^b^	0^a**^	0^a**^
									3.45413^b^			
60 µg/ml MPR	0^a^	0.6667 ± 0.1878^a^	0.0333 ± 0.0333^a*^	0.3667 ± 0.1221^a^	0.2 ± 0.1213^a^	0^a**^	0^a**^	0.0333 ± 0.03333^a*^	1.6333 ±	0^a**^	0^a**^	0^a**^
									0.30127^a^			
600 µg/ml MP	0.4333 ± 0.1837^a^	1.5 ± 0.2702^a^	0^a**^	1.6667± 0.4657^a^	0.1667 ± 0.1363^a^	0^a**^	0^a**^	0^a**^	0.4 ±	0^a**^	0^a**^	0^a**^
									0.25641^a^			
600 µg/ml MPR	1.6667 ± 0.4557^b^	2.0333 ± 0.3474^a^	0.0333 ± 0.0333^a*^	0.1 ± 0.1^a^	0.0333 ± 0.0333^a*^	0^a**^	0^a**^	0.0667 ± 0.06667^a^	0.5 ± 0.5^a^	0.0333 ± 0.03333^a*^	0^a**^	0^a**^

After 15 days of recovery under normal conditions, it was clear that with the 6 μg/ml MPs, there was a marked improvement in the teardrop and boat-shaped cells, while the schistocytes, helmet cells, ovalocytes, keratocytes, SC poikilocytes (poikilocytes are cells that have a variable appearance but are usually dense and may resemble sickle cells. They often have single or multiple angulated branches; some of which that resemble sickle cells may instead have straight edges. Classical SC poikilocytes may be quite rare, so they must be actively sought), and sickle cell types had completely disappeared. The recovery rate was 10.24%. With the 60 μg/ml MPs, there was a marked improvement in teardrop-like cells, helmet cells, ovalocytes, and echinocytes, while the boat-shaped, triangular, and folded shape cells and stomatocyte types had completely disappeared. The recovery rate was 15.63%. With the 600 μg/ml MPs, there was a marked improvement in the helmet cell types and ovalocytes, with a significant increase in the number of boat-shaped cells, teardrop-shaped cells, sickle cells, and echinocytes. The response rate of the animals to recovery appeared at the concentration of 109.6%, and this does not mean that the recovery rate here is as high as would be expected due to the continued high numbers of abnormal blood cells. This is evidence of the presence of MPs and that the animals could not eliminate the high concentration of MPs inside their bodies. Considering the preceding, we conclude that a 15-day recovery period helped mitigate the impacts of microplastics in small quantities but was insufficient to eliminate them; however, at the same time, in high concentrations, it was never sufficient (Table 2).

**TABLE 2:** Response of the animals after 15 days of recovery.

Treatment	Total number of exposure alteration	Total number of recovery alteration	Animal response for recovery (%)
C	285	107 ↓	37.54
6 MP	859	88 ↓	10.24
60 MP	563	88 ↓	15.63
600 MP	125	137 ↑	109.60

**FIGURE 4:**
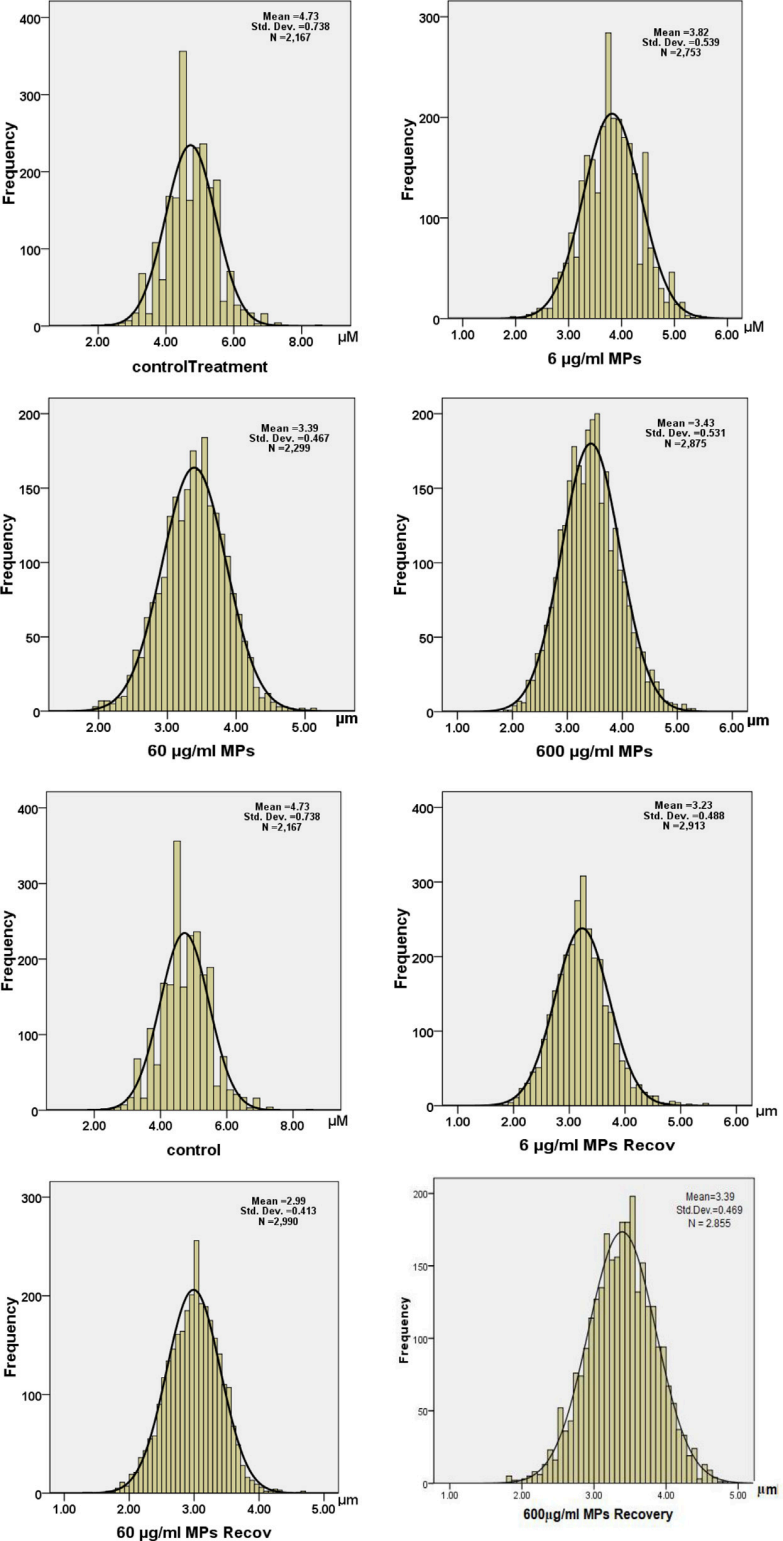
Histogram of diameters of erythrocytes in the exposure and recovery groups of the C57BL/6 murine model.

Moreover, a drop in the RBC diameter indicates several types of blood anemia. In this study, the diameter of the RBCs was measured in all animal groups, showing a significant decrease in the MPs-treated groups: 3.8, 3.3, and 3.4 μm for 6, 60, and 600 μg/ml MPs, respectively (Figure 4). Together, the aforementioned studies have demonstrated how altered RBC shape transitions might be impacted by lower RBC deformability.

### 3.3 Hematological parameters

In the treated groups, the concentration of 6 µg/ml MPs affected (p> 0.05) neutrophils and lymphocytes; the concentration of 60 µg/ml MPs significantly affected (p < 0.05) RBCs, Hbs, HTs, and monocytes; these concentrations had highly significant effects (o < 0.00001) on neutrophils, lymphocytes, and the N/L ratio. By contrast, the concentration of 600 µg/ml MPs was found to be significant on monocytes only and highly significantly on RBCs, Hbs, HTs, neutrophils, lymphocytes, and the N/L ratio (Table 3). Only the RBCs, HCT, Hbs, neutrophils, lymphocytes, monocytes, and N/L ratio remained affected by MPs as stressed by the treatment of 60 and 600 μg/ml MPs during the recovery periods. The MPs-exposed animals’ hematological parameters showed substantial fluctuations when compared to those of the control and of 6 μg/ml MPs group. Except for RBCs and HCT, which showed substantial alterations after recovery from the pollutant, there was no other significant variation in the hematological parameters. In contrast to the exposed animals, these changes may have been brought about by individual differences. In conclusion, the concentration of MPs plays a significant role in determining how harmful these are, and the 15-day recovery time improved the hematological features.

**TABLE 3:** Effect of 15 days of exposure to microplastics (MPs) and recovery on the hematological characteristics of the C57BL/6 mouse model. Data are represented as means ± SE. Values with different superscript letters in the same row for each parameter are significantly different (*p* < 0.05). Values of recovery period with * are significantly different from exposure period.

Hemato-biochemical parameters	Exposure period				Recovery period			
	Control	6 µg	60 µg	600 µg	Control	6 µg	60 µg	600 µg
RBCs	9.873±0.339^b^	9.313±0.330^b^	8±0.196^a*^	7.625±0.209^a**^	9.865±0.171^b^	9.7075±0.3284^b^	9.458±0.1777^b^	8.35±0.050^a*^
Hb	14.425±0.708^c^	13.05±0.695^b^	11.475±0.213^ab*^	10.135±0.404^a**^	14.675±0.4768^c^	14.245±0.4211^b^	12.893±0.4809^b*^	8.35±0.050^a**^
Ht (PCV)	54.45±1.945^b^	51.725±2.164^bc^	47.875±1.245^b*^	40.925±0.927^a**^	55.4±1.064^b^	53.05±2.5105^b^	54.575±0.669^b^	44.85±1.350^a*^
MCV	55.355±2.849^a^	55.668±2.513^a^	60.05±2.944^a^	2001.5±1515.313^a^	51.72±5.649^a^	54.715±2.5125^a^	57.775±1.4008^a^	53.725±1.935^a^
MCH	14.645±0.760^a^	14.08±1.001^a^	14.368±0.137^a^	9.075±0.118^a^	14.875±0.446^a^	14.69±0.264^a^	13.638±0.4835^a^	13.53±0.200^a^
MCHC	26.463±0.636^a^	25.205±0.804^a^	24.038±0.969^a^	56.25±1.314^a^	26.4775±0.635^a^	26.9525±0.8251^a^	23.655±1.0297^a^	25.215±0.535^a^
Platelets	717.75±65.288^a^	557±40.484^a^	557.5±38.291^a^	41±0.912^a^	712.75±3.355^a^	2.35778±1.736^a^	5.715±2.4295^a^	521.5±3.250^a^
WBCs	8.375±0.327^a^	8.425±0.335^a^	8.325±0.175^a^	9.075±0.118^a^	7.7±0.0913^a^	8.2±0.339^a^	7.825±0.317^a^	8.5±0.1000^a^
Neutrophils	31.5±0.288^a^	36.75±0.479^b*^	46.75±0.853^c**^	56.25±1.314^d**^	31.5±0.289^a^	31.25±0.250^a^	35±0.4083^b**^	41±0^c**^
Lymphocytes	63.25±0.25^d^	59.25±0.479^c*^	51±0.912^b**^	41±0.912^a**^	63±0.358s^c^	63.5±0.2887^c^	60.25±0.25^b*^	55.5±0.50^a**^
Eosinophils	0.75±0.25^a^	0.75±0.25^a^	0.5±0.288^a^	0.75±0.25^a^	1±0	1±0	1±0	1±0
Basophils	0.5±0.288^a^	0.5±0.288^a^	0±0^a^	0.75±0.25^a^	1±0^a^	1±0^a^	1±0^a^	1.5±0.500^a^
Monocytes	4±0.408^b^	4±0.408^ab^	1.75±0.479^a*^	1.75±0.479^a*^	3.5±0.289^b^	3.25±0.250^b^	2.75±0.250^b^	1±0^a**^

### 3.4 Biochemical parameters

The metabolic profile of the animals exposed to MPs changed, and the liver enzyme activity, particularly elevated aspartate aminotransferase (AST) and alanine aminotransferase (ALT) (Connes, Lamarre, et al.), increased considerably with the dose. A comparable pattern of the considerable rise in serum glucose was seen. When comparing the treated animals with those of the control group, the treated animals’ creatinine levels were higher. Animals exposed to MPs displayed noticeably higher levels of total protein. Additionally, a considerable rise in the neutrophil to lymphocyte ratio (N/L), a reliable immunological indicator of inflammation, was dose-dependently seen in the MPs-treated mice (Table 4).

**TABLE 4:** Effect of 15 days of exposure to microplastics (MPs) and recovery on the biochemical parameters of the C57BL/6 murine model. Data are represented as means ± SE. Values with different superscript letters in the same row for each parameter are significantly different (*p* < 0.05). Values of recovery period with * are significantly different from exposure period.

Hemato-biochemical parameters	Exposure period				Recovery period			
	Control	6 µg	60 µg	600 µg	Control	6 µg	60 µg	600 µg
AST	80±5.115^a^	80±5.115^a^	95.5±3.014^ab*^	109.5±2.629^b**^	77.5±0.86603^a^	77.5±0.86603^a^	87.5±1.84845^b*^	86.5±1.500^b*^
ALT	43±2.708^a^	43±2.708^b*^	62.75±1.887^c**^	65±-0.816^c**^	44.5±0.9574^a^	41.25±1.65202^a^	54.25±0.62915^b*^	61.5±3.500^c**^
Glucose	138±3.582^a^	138±3.582^a^	155.25±6.725^ab^	162±2.345^b*^	1.2525±2.28674^a^	1.33±5.14782^a^	132.25±1.18145^a^	1.385±3.500^a^
Total protein	4.775±0.154^a^	4.775±0.1547^ab^	5.45±0.05^bc*^	5.925±0.137^c**^	4.275±0.08539^a^	4.575±0.08539^a^	5±0.09129^b**^	5.25±0.0500^b**^
Creatinine	0.375±0.047^a^	0.375±0.0478^a^	0.525±0.047^ab^	0.7±0.040^b*^	0.325±0.04787^a^	0.375±0.02500^a^	0.45±0.02887^ab^	0.55±0.0500^c*^
N\L ratio	0.497±0.0047^a^	0.4975±0.004^a^	0.9175±0.033^b**^	1.3775±0.0614^c**^	0.5003±0.00917^a^	0.4922±0.00555^a^	0.581±0.00880^b**^	0.7388±0.7388^c**^

## 4 Discussion

In recent years, a significant increase in the negative impact of environmental contaminants on human health (Inhorn and Patrizio. 2015) is seen. High MP concentrations have been found in freshwater (0-1 106 items/m^3^) and marine (0-1 104 items/m^3^) waterbodies. MPs have also been seen in various animal species such as mussels and fish that humans consume (Desforges et al., 2014; Deng et al., 2017). MPs can spread through the aquatic food chain, which will likely cause a biological buildup of the substance. Microplastics (MPs), an environmental pollutant, cause toxicity in the liver, kidneys, and gastrointestinal system (Hou et al., 2021; Wang et al., 2021; Zhao et al., 2021) in animals and aquatic organisms. A recent study has shown that MPs harm the male reproductive system (Hou et al., 2021). However, little is known regarding how microplastics affect vascular biology or humans/mammals. A recent study has revealed that MPs fundamentally impact the hematological system in mice and that these alterations in gene expressions were connected (Sun et al., 2021). However, further research is needed to determine how MPs impact blood cells and other hematological variables. Red blood cell deformability has a significant impact on blood circulation at the microcirculation level. Consequently, any reduction in RBC deformability could impact flow resistance, tissue perfusion, and oxygenation (Nader et al., 2019). In this investigation, we discovered that MP particle accumulation was primarily dose dependent in the gastrointestinal system. Their particle size highly influences their distribution and the tissue accumulation kinetics, and they accumulate in the liver, kidneys, and gut (Deng et al., 2017). The cellular components in the plasma—an aqueous solution which includes organic compounds, proteins, and salts—make up the whole blood, a two-phase liquid (Baskurt and Meiselman 2003). The blood’s erythrocytes, leukocytes, and platelets make up its cellular phase. The white blood cells and platelets impact blood rheology, but under typical circumstances, erythrocytes (RBCs) have the most significant impact (Pop et al., 2002) The physical characteristics of these two phases and their proportional contributions to the total blood volume determine the rheological characteristics of the blood. Additionally, hematocrit, plasma viscosity, RBCs’ capacity to deform under flow, and RBC aggregation–disaggregation characteristics all affect blood viscosity (Baskurt and Meiselman 2003; Cokelet et al., 2007).

Teardrop-like cells (Pollastro and Pillmore, 1987), helmet cells (HE), stomatocytes (Sto), sickle cells (Sic), schistocytes (Sch), folded cells (Gregorio et al., 2009), boat-shaped cells (Bo), ovalocytes Ov), and echinocytes are only a few of the RBC alterations that have been observed in the groups exposed to MPs (Lechner and Ramler). Teardrop-like cells (Pollastro and Pillmore, 1987), which are thought to be a marker for myelofibrosis, significantly increased in the 6 μg/ml MPs and 60 μg/ml MPs groups before reducing on their own after 15 days. The echinocytes are spiculated RBCs in high numbers in the 60 μg/ml MPs blood samples and indicate widespread electrolyte depletion. The distortion of red blood cells into echinocytes is due to the bilayer membrane alterations, which is the result of a protective mechanism (Svetina 2012). On the other hand, there is another explanation mechanism as high ROS levels can easily promote lipid peroxidation because RBC membranes contain a lot of polyunsaturated fatty acids, which further damages RBCs by disrupting membrane integrity and lowering their resistance to injury (Remigante et al., 2022). It is possible to utilize the cationic surfactant benzalkonium chloride as a cell membrane surface state changer since it can integrate into the erythrocyte membrane and change the shape of the erythrocytes in saline solution (Rudenko and Saeid 2010).

A reduction in intracellular erythrocyte potassium (K+) leads to red blood cell dehydration and echinocyte formation (Glader and Sullivan 1979; Gallagher 2017). Most of these alternations in the 6 μg/ml MPs and 60 μg/ml MPs groups were recovered after 15 days, demonstrating the blood’s quick, natural mending process. The recovery time, however, was insufficient for the 600 μg/ml MPs group to return the RBCs to their characteristic morphology. Thus, a 15-day recovery period helped lessen the impacts of microplastics in lower dosages but was insufficient to eliminate them; by contrast, it was never adequate at high concentrations (Table 4). The discovered RBC alternations impacted normal blood flow because they affected their capacity for deformation (Figure 3C) and subsequently led to the formation of cellular aggregations.

Blood viscosity is linearly connected to hematocrit since it depends on the quantity (and number) of erythrocytes in the blood (Nader et al., 2019). At low shear rates (such as in the veins), hematocrit (HT) has a more significant effect on blood viscosity than it does at high shear rates (such as in the arteries) (Cokelet et al., 2007). According to estimates, a one unit increase in hematocrit at high shear rates would result in a 4% increase in blood viscosity (if RBC rheological properties remain the same). In the current investigation, the 60 and 600 μg/ml MPs groups had considerably lower HT levels. Additionally, at the higher MP concentrations (60 and 600 μg/ml MPs), hemoglobin (Hb) levels were much lower, which is thought to be an indication of sickle cell anemia (Magrì et al., 2018). Additionally, RBCs become more fragile and susceptible to hemolysis when they lose their deformability (Connes et al., 2014). Vaso-occlusion and pre-capillary obstruction can both be caused by stiff, sickle-shaped RBCs (Rees et al., 2010).

After exposure to MPs, the biochemical markers (creatinine, AST, ALT, glucose, and total protein) significantly increased. Creatinine can be used as a biomarker for renal impairment and as an indicator of glomerular filtration rate (Lien et al., 2006).

Enzymes (AST and ALT) are found in the cells of several organs throughout the body (Lenaerts et al., 2005). These enzymes’ release and increased blood levels are signs of damaged cell membranes (Lenaerts et al., 2005). According to our findings, paraquat and/or microplastic particles increased the activity of intracellular enzymes (ALT and AST), which may be a sign that cell plasma membranes have been damaged (Cheng et al., 2022). Proteins are essential for preserving physiological homeostasis and in stopping blood leak from the circulatory system (Bergmeier and Hynes 2012). Increased levels of total protein result from MP exposure, and these suggest problems in the kidney and liver functions.

With low MP dosages, when compared to the control group, microplastics buildup and hemato-biochemical changes were improved after the recovery period. However, the high dose group was affected negatively by MPs. The recovery period has an effect ranging from cells to tissues where the defense mechanisms were reported (Hamed et al., 2019; Hamed et al. 2020; Hamed et al. 2021; Sayed et al., 2021; Ammar et al., 2022; Sayed et al., 2022; Sayed et al., 2022).

## 5 Conclusion

In C57BL/6 mice, microplastics produced a range of toxic consequences, which included anemia and changes in hemato-biochemical parameters, which may induce severe toxic effects in all organs at higher concentrations and for extended periods. Our findings have shown that MPs had damaging effects on mice’s RBCs, reflecting the dangerous implications of these MPs on human health. The current study may initiate future comprehensive studies on the impacts of MPs on other body systems.

## Data Availability

The raw data supporting the conclusions of this article will be made available by the authors, without undue reservation.
